# Cancer biomarkers and targeted therapies

**DOI:** 10.1186/2045-3701-3-6

**Published:** 2013-02-06

**Authors:** Zhiyuan Shen

**Affiliations:** 1 The Cancer Institute of New Jersey, Robert Wood Johnson Medical School, 195 Little Albany Street, New Brunswick, NJ 08903, USA

## 

Cancer is a disease that showcases great molecular diversity among many individuals. Patients with the same clinically diagnosed cancer often vary in their response to the same treatment because each case of cancer has a unique molecular signature and pathological evolution. Therefore, individualized therapy is paramount for improving of cancer treatment. The development of rationalized and individualized therapy is reliant on the identification of the specific biomarkers, validation of the biomarkers to identify the therapeutic targets, and drug development against the identified targets.

Cancer biomarkers are the measurable molecular changes to either cancerous or normal tissues of patients. Although the term “biomarker” most commonly refers to altered expression of certain gene products or abnormal DNA configurations, changes to cellular processes such as energy metabolism and DNA damage response can also be used as biomarkers in a broader sense. Cancer biomarkers have multiple implications in cancer intervention. A reliable biomarker can be used for cancer diagnosis, risk and prognosis assessments, and for the surveillance of treatment effectiveness. More importantly, some, but not all, biomarkers can be exploited as therapeutic targets. This is because some biomarkers may be simply “messengers” that do not directly contribute to the tumor growth and are thus not ideal therapeutic targets. Only the “driver” or “conspirator” biomarkers that directly contribute to tumor growth may be targeted for therapy. Therefore, effort in the development of targeted therapies must not simply to identify biomarkers, but also to understand the biological significance of such markers in order to validate their usefulness as potential therapeutic targets. In this special issue of *Cancer Biomarkers and Targeted Therapies,* we present 5 reviewers that offer a glimpse of the various topics in cancer biomarker and targeted therapy research.

A universal cancer biomarker is the Warburg effect, the shift of mitochondrial energy production to a glycolysis dependent metabolism that provides not only energy for cells but also produces intermediate building materials for cancer cells to grow. Multiple regulators control this switch of energy metabolism. The article by Liang et al. summaries the current understanding on how the tumor suppressor gene p53 controls the cellular energy metabolism [1].

Altered DNA repair capabilities are considered a functional biomarker. Like normal cells, cancer cells encounter various forms of endogenous and exogenous DNA damage. Proper functions of multiple DNA repair pathways are essential for cancer cells to sustain their growth and resist therapeutic DNA damage. Defective DNA repair not only contributes to genomic instability and tumorigenesis [2], but also offers opportunity for targeted therapy. It is known that some cancers with defects of a primary DNA repair pathway may be reliant on an alternative or backup DNA repair pathway(s). This offers a possibility to target the alternative DNA pathways, which would confer synthetic lethality to the cancer cells harboring the original repair defect. The articles by Santivasi and Xia [3] and by Zhang [4] in this special issue discuss how to exploit the distinct roles of homologous recombination and non-homologous end joining in DNA double strand break repair and the function of homologous recombination in DNA single strand break repair for targeted therapies.

In addition, two papers in this special issue discuss the roles of specific genes in the context of cancer progression and therapy. The paper by Yue et al. [5] discusses the pros and cons of using cytoskeleton protein filamin-A as a cancer biomarker and potentially a therapeutic target. Lastly, the paper by Allaj et al. [6] summarizes the roles of cyclooxygenase and prostanoid signaling in cancer progression and as therapeutic target for the treatment.

Due to the broad nature of cancer biomarkers and targeted therapies, it is not our intention to cover all major aspects of this active research area in a single issue. However, we hope that these articles offer readers a flavor of how alternations of specific genes, DNA damage response, and energy metabolism may be used as cancer biomarkers and for targeted therapies.
